# Association between inter-arm blood pressure difference and cardiovascular disease: result from baseline Fasa Adults Cohort Study

**DOI:** 10.1038/s41598-023-36205-1

**Published:** 2023-06-14

**Authors:** Mehdi Sharafi, Zahra Amiri, Elham Haghjoo, Sima Afrashteh, Siavash Dastmanesh, Maryam Talebi Moghaddam, Azizallah Dehghan, Helma Al-Sadat Tabibzadeh, Ali Mouseli

**Affiliations:** 1 Noncommunicable Diseases Research Center, Fasa University of Medical Sciences, Fasa, Iran; 2 Social Determinants in Health Promotion Research Center, Hormozgan Health Institute, Hormozgan University of Medical Sciences, Bandar Abbas, Iran; 3 Department of Persian Medicine, Fasa University of Medical Sciences, Fasa, Iran; 4 Department of Biostatistics and Epidemiology, Faculty of Health and Nutrition, Bushehr University of Medical Sciences, Bushehr, Iran; 5 Department of Sport Sciences, Abadeh Branch, Islamic Azad University, Abadeh, Iran; 6 Department of Biostatistics and Epidemiology, School of Health, Kerman University of Medical Sciences, Kerman, Iran; 7 Student Research Committee, Fasa University of Medical Sciences, Fasa, Iran

**Keywords:** Cardiology, Diseases, Health care, Medical research, Risk factors

## Abstract

The inter-arm blood pressure difference has been advocated to be associated with cardiovascular mortality and morbidity. Our study aimed to investigate the association between Inter-arm systolic and diastolic blood pressure differences and Cardio Vascular Disease (CVD). A total of 10,126 participants aged 35–70 years old were enrolled in a prospective Fasa Persian Adult Cohort. In this cross-sectional study, the cutoff values for inter-arm blood pressure difference were less than 5, greater than 5, greater than 10, and greater than 15 mm Hg. Descriptive statistics and logistic regression were used to analyze the data. Based on the results the prevalence of ≥ 15 mmHg inter-arm systolic and diastole blood pressure difference (inter-arm SBPD and inter-arm DBPD) were 8.08% and 2.61%. The results of logistic regression analysis showed that inter-arm SBPD ≥ 15 and (OR_<5/≥15_ = 1.412; 95%CI = 1.099–1.814) and inter-arm DBPD ≥ 10 (OR_<5/≥10_ = 1.518; 95%CI = 1.238–1.862) affected the risk of CVD. The results showed that the differences in BP between the arms had a strong positive relationship with CVD. Therefore, inter-arm blood pressure could be considered a marker for the prevention and diagnosis of CVD for physicians.

## Introduction

Systolic and diastolic blood pressure are important risk factors for CVD^1^. According to the latest guidelines for managing high blood pressure, blood pressure should be measured in both arms^2^. Inter-arm blood pressure difference can be easily measured in outpatient clinics^3^, and in most cases, there is a difference between the blood pressure of each arm^4^. 10 mmHg and above is defined as an increase in inter-arm SBPD difference^5^ and it can be found in more than 24% of healthy people^6^. Detection of this difference is important for diagnosing and treating hypertension^7^. The prevalence of inter-arm blood pressure difference varies in different populations and this difference tends to increase in the presence of high blood pressure^8^. The inter-arm blood pressure difference is reported in 7% of diabetic patients, and 11% in high blood pressure patients^9^. In a study conducted by Essa et al., inter-arm systolic blood pressure difference greater than 10 mmHg was observed in 26.1% of study participants^3^. In addition, this difference is more prevalent in those with a history of high blood pressure^2^. The results of the study conducted by Sharma et al. showed that 46% of the participants had a systolic blood pressure difference of 10 mmHg and more^10^. Based on previous studies, there is a positive and significant relationship between the inter-arm blood pressure difference and the occurrence of cardiovascular diseases^11,12^. In Iran, few studies with sufficient sample size have investigated the relationship between the difference in inter-arm systolic and diastolic blood pressure and the occurrence of cardiovascular diseases. Therefore, this study aimed to determine the relationship between Inter-arm systolic and diastolic blood pressure differences and CVD in Iran.

## Methods

### Study population

The current research data is related to Fasa Adults Cohort Study (FACS) data on 10,126 residents of Sheshdeh City, which is located 40 km from Fasa City. This longitudinal study is one of 22 ongoing cohorts in Iran, which is based on the population and aims to investigate the risk factors of cardiovascular diseases.

### Inclusion and exclusion criteria

The criteria for inclusion in the study are people over 35 to 70 years of age who have lived in the Shashada and Qarabalag region for at least 10 years and have consent to participate in the study, the exclusion criteria are people unable to answer questions and mentally and physically disabled.

### Data collection

People were invited to participate in the study by telephone through a list that included people covered by each health center and provided to the staff. An average of 25 people were invited to the cohort center daily and demographic information, job type, physical activity level, information related to anthropometric examination (height, weight, waist circumference, hip circumference), medical examination including oral and dental hygiene, blood pressure and the history of infectious and non-infectious diseases, biological samples including blood, hair, urine, and feces were recorded.

### Measurements

Demographic information and socioeconomic status of people were collected by general interviewers, biological samples were collected by laboratory experts, and medical information was collected by five trained nurses. Resting heart rate and blood pressure were measured using calibrated devices in both arms consecutively after 15 min of rest and with a time interval of 5 min, and its average was recorded. Other medical information including the history of infectious and non-infectious diseases; Oral and dental hygiene and anthropometric measurements including (height, weight, waist circumference, and hips; calculation of body mass index (BMI) and duration of sleep in 24 h).

In this cross-sectional study, the difference in systolic and diastolic blood pressure between the two arms was calculated, and individuals were grouped into 4 groups with blood pressure differences of less than 5, greater than 5, greater than 10, and greater than 15 mm Hg. Finally, the relationship between these groups of cardiovascular diseases was evaluated.

### Outcome

Dependent variable: having cardiovascular diseases except for high blood pressure.

### Independent variable

Systolic and diastolic blood pressure differences between the two arms.

#### Covariates

Gender, age, triglyceride glucose (TyG), triglyceride to high-density lipoprotein cholesterol (TG-HLD-C), smoking, opium use, diabetes, triglyceride (TG: mg/dl), high-density lipoprotein (HDL-c: mg/dl), depression, socioeconomic status, BMI (kg/m^2^), waist circumference (WC), and non-alcoholic fatty liver.

### Statistical analysis

Descriptive statistics were reported in the form of a number (percentage) or mean ± standard deviation based on the presence or absence of CVD. An independent t-test was used to compare quantitative variables between the two groups, and a chi-score was used to examine the relationship between qualitative covariates and cardiovascular disease. Multiple logistic regression was used to investigate the relationship between the difference in systolic and diastolic blood pressure between the two arms and having cardiovascular diseases and to control the effect of other study covariates. All analyses consider a level of significance below 0.05. STATA version 14.0 (Stata Corporation, College Station, TX, USA). was used to analyze the data.

### Ethics approval and consent to participate

Ethical issues including plagiarism, informed consent, misconduct, data fabrication and/or falsification, double publication and/or submission, redundancy, etc. were completely observed by the authors. This study was performed according to the ethical guidelines expressed in the Declaration of Helsinki and the Strengthening of the Reporting of Observational Studies in Epidemiology (STORB) guideline. The study was also approved by the Research Ethics Committee of Fasa University of Medical Sciences (IR.FUMS.REC.1401.144). Informed consent was also waived by the Research Ethics Committee of Fasa University of Medical Sciences (IR.FUMS.REC.1401.144).

## Result

The prevalence of CVD in this study was 11.73%. Of the 10,126 participants in the study, 5283 (52.17%) were female. The majority of patients (766, 64.48%) were identified in the inter-arm SBPD < 5 mmHg group, followed by 96 (8.08%) patients who belonged to the inter-arm SBPD group ≥ 15 mm Hg. Also, inter-arm DBPD ≥ 10 mm Hg was reported in 146 (12.29%) patients. A few 31 (2.61) patients were inter-arm DBPD ≥ 15. The mean age (55.55 ± 9.17 years) and body mass index (26.47 ± 4.85) were raised in those with CVD. There were 443 (9.69%) male patients and 745 (13.41%) female patients. Out of the total (1188, 11.73%) who had cardiovascular disorders, 289 (24.33%) and 189 (15.91%) patients presented with diabetes and Non-alcoholic fatty liver. Furthermore, people with depression are at increased risk of CVD compared to healthy (P < 0.05) (Table 1).

**Table 1:** Patient characteristics of the study population at baseline by having cardiovascular diseases.

Variables	Subgroup	Cardiovascular diseases		P-value
		No, N (%)	Yes, N (%)	
Gender	Male	4130 (90.31)	443 (9.69)	< 0.001*
	Female	4808 (86.59)	745 (13.41)	
Age	Mean ± SD	47.71 ± 9.24	55.55 ± 9.17	< 0.001**
Socioeconomic status	Low	2898 (32.44)	477 (40.19)	< 0.001*
	Middle	2977 (33.32)	399 (33.61)	
	High	3059 (34.24)	311 (26.20)	
Inter-arm SBPD	** < **5	6074 (67.97)	766 (64.48)	< 0.001*
	≥ 5	1466 (16.41)	163 (13.72)	
	** ≥ **10	981 (10.98)	163 (13.72)	
	** ≥ **15	415 (4.64)	96 (8.08)	
Inter-arm DBPD	** < **5	6896 (77.17)	878 (73.91)	< 0.001*
	≥ 5	1178 (13.18)	133 (11.20)	
	** ≥ **10	686 (7.68)	146 (12.29)	
	** ≥ **15	176 (1.97)	31 (2.61)	
BMI		25.53 ± 4.84	26.47 ± 4.85	< 0.001**
Waist circumference		92.74 ± 11.74	96.23 ± 11.72	< 0.001**
Quantile TyG index	Q1	2335(26.13)	195(16.43)	< 0.001*
	Q2	2235(25.01)	295(24.85)	
	Q3	2214(24.78)	317(26.71)	
	Q4	2151(24.07)	380(32.01)	
Quantile TG-HLD-c ratio	Q1	2290(25.67)	235(19.81)	< 0.001*
	Q2	2230(25.00)	298(25.13)	
	Q3	2175(24.38)	352(29.68)	
	Q4	2226(24.95)	301(25.38)	
Smoking	No	6530(73.06)	840(70.71)	0.087*
	YES	2408(26.94)	348(29.29)	
Opium addiction	No	7051(78.89)	963(81.06)	0.083*
	YES	1887(21.11)	225(18.94)	
Diabetes	No	7979(89.27)	899(75.67)	< 0.001*
	YES	959(10.73)	289(24.33)	
TG		130.79 ± 82.51	138.07 ± 82.51	0.004**
HDL-c		50.88 ± 16.08	51.25 ± 15.32	0.451**
Total sleep in 24 h		7.78 ± 1.85	7.52 ± 1.92	< 0.001**
Depression	No	8376(93.71)	1070 (90.07)	< 0.001*
	YES	562(6.29)	118(9.93)	
Fatty liver	No	8081(90.41)	999(84.09)	0.034*
	YES	857(9.59)	189(15.91)	

*SBPD* systolic blood pressure difference, *DBPD* diastolic blood pressure difference.

*p-value of chi-square test.

**p-value for t-test.

**Figure 1:**
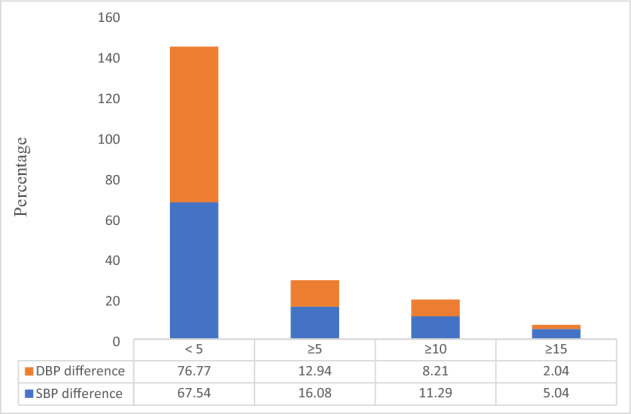
Prevalence of inter-arm blood pressure difference among study subjects.

Regarding the prevalence of inter-arm BP differences in total subjects, it was found that 12.94% (n = 1144) had a difference of ≥ 10 mm Hg (systolic BP), and about 5.04% (n = 511) were at high risk for CVD events (difference ≥ 15 mmHg). Regarding the difference in diastolic BP for both arms, it was observed that 8.21% (n = 832) had a difference of ≥ 10 mm Hg and where as 2.04% (n = 207) had a difference of ≥ 15 mm Hg. (Fig. 1).

Table 2 shows the association between inter-arm SBPD and CVD in adults. There was a significant positive association found between SBPD ≥ 15 and CVD (OR_<5/≥15_ = 1.412; 95%CI = 1.099–1.814; P = 0.007), while there was no significant association found between inter-arm SBPD ≥ 10 (OR_<5/≥10_ = 1.148; 95%CI = 0.946–1.394; P = 0.160) and inter-arm SBPD ≥ 5 (OR_<5/≥5_ = 0.920; 95%CI = 0.763–1.110; P = 0.385) with CVD.

**Table 2:** Multiple logistic regression for evaluating the association between inter-arm systolic blood pressure difference (inter-arm SBPD) and cardiovascular diseases.

Variable	Subgroup	Adjusted odds ratio^a^	Std. error	95% CI for odds ratio	P-value
Inter-arm SBPD	** < **5	1	–	–	–
	≥ 5	0 .920	0 .087	0 .763 to 1.110	0.385
	** ≥ **10	1.148	0.113	0.946 to 1.394	0.160
	** ≥ 15**	**1.412**	0 .180	1.099 to 1.814	**0.007**

^a^Odds ratio adjusted for sex, age, BMI, socioeconomic status, waist circumference, TyG index, quantile TG-HLD-c ratio, smoking, opium addiction, diabetes, total sleep, depression, and fatty liver.

Significant values are in bold.

Table 3 shows the association between inter-arm DBPD and CVD in adults. There was a significant positive association found between inter-arm DBPD ≥ 10 and CVD (OR_<5/≥10_ = 1.518; 95%CI = 1.238–1.862; P < 0.001), while there was no significant association found between inter-arm DBPD ≥ 15 (OR_<5/≥15_ = 1.218; 95%CI = 0.806–1.840; P = 0.347) and inter-arm DBPD ≥ 5 (OR_<5/≥5_ = 0.872; 95%CI = 0.713–1.068; P = 0.187) with CVD.

**Table 3:** Multiple logistic regression for evaluating the association between inter-arm diastolic blood pressure difference (inter-arm DBPD) and cardiovascular diseases.

Variable	Subgroup	Adjusted odds ratio^a^	Std. error	95% CI for odds ratio	P-value
inter-arm DBPD	** < **5	1	–	–	–
	≥ 5	0.872	0.089	0.713 to 1.068	0.187
	** ≥ 10**	**1.518**	**0.158**	**1.238 to 1.862**	** < 0.001**
	≥ 15	1.218	0.256	0.806 to 1.840	0.347

^a^Odds ratio adjusted for sex, age, BMI, socioeconomic status, waist circumference, TyG index, quantile TG-HLD-c ratio, smoking, opium addiction, diabetes, total sleep, depression, and fatty liver.

Significant values are in bold.

## Discussion

Hypertension is a well-known non-communicable disease that has received public health attention. High blood pressure screening using a blood pressure measuring device is one of the simplest and easiest methods to identify people with high blood pressure. However, the concept of measuring blood pressure in both arms to identify the difference in blood pressure has not yet been considered in public health. It is necessary to identify people who have a significant difference in blood pressure between the two arms to prevent cardiovascular diseases^2^ .

In the previous cohort studies, the inter-arm blood pressure difference was determined by consecutive measurements rather than simultaneous measurements. By inflating the cuff on one side during measuring blood pressure, blood pressure increases in the opposite arm^13^ Therefore, consecutive measurement of blood pressure in each arm with a Sphygmomano is not correct. As a result, in recent studies, the difference in blood pressure between the arms is measured simultaneously with an automatic oscillometric device^14^ Accordingly, it is preferred to measure blood pressure simultaneously from both arms to obtain the blood pressure difference between the arms.

In this study, the prevalence of inter-arm SBPD is reported to be 10 mm Hg and more than in 43.5% of participants. Also, a diastolic blood pressure difference of 5 mmHg and more was observed in 57.2% of them. Due to the high prevalence of the blood pressure difference between the arms, it should be considered to measure blood pressure in both arms. These people are at risk of developing cardiovascular diseases in the future, so they need to be observed and followed up^2^ The results of the Seethalakshmi et al. study showed that 46% of the participants had an inter-arm SBPD of 10 mmHg or more and 35% of them had an inter-arm DBPD of 5 mmHg or more^15^ . Additionally, another study showed that 26.1% of the participants had an inter-arm SBPD of 10 mmHg or more^16^ . According to the studies, the difference in systolic blood pressure between the two arms is more observed in the elderly^17,18^ . Several studies have reported that in patients with inter-arm blood pressure differences, the blood pressure in the right arm is higher than in the left arm, which may be related to the anatomical difference in the left and right blood circulation^19^ .

The results of this study indicated that the inter-arm SBPD and DBPD between the arms was 15 mm Hg and more in 5.04% and 2.04% of the participants, respectively. According to the study of Kranenburg et al., 16% of the participants had an inter-arm systolic blood pressure difference of 15 mmHg or more^20^ .

In this study, the prevalence of cardiovascular diseases in women was significantly higher than in men. Based on current knowledge, it seems that the risk of cardiovascular diseases in women is increased compared to men due to factors such as diabetes, blood pressure, and obesity. Also, the occurrence of these diseases in women is influenced by socioeconomic and psychosocial factors. Therefore gender aspects should be more intensively considered^21^.

The present study showed a significant relationship between substance use and cardiovascular disease, which is consistent with the results of other studies^22,23^. Long-term drug use leads to changes in plasma fibrinogen levels, coagulation, atherosclerosis, development or exacerbation of coronary artery disease (CAD), hypertension, cardiac arrest, and stroke^24^.In this study, an inter-arm DBPD of more than 10 mmHg was associated with CVD, while an inter-arm DBPD of more than 15 mmHg was not. But with the increase in the inter-arm SBPD between the arms, the prevalence of CVD increased. The results of previous studies show that recently there is a significant difference between inter-arm SBPD and cardiovascular outcomes^20,25^ . The results of the meta-analysis study conducted by Clark et al. suggested that a blood pressure difference of 10 mmHg and more or 15 mm Hg and more is related to peripheral vascular disease^26^. Peripheral vascular disease has been identified as a risk factor for cardiovascular events and mortality due to it^27^. and based on studies conducted, peripheral vascular disease is the cause of the inter-arm SBPD between the two arms^28^. Also, it is notable that, a difference greater than 15 mm Hg is associated with evidence of carotid angiography or aortic artery disease and vascular disease caused by high blood pressure^8^. In addition, results from multiple logistic regression analysis demonstrated that inter-arm SBPD > 10 mm Hg was positively related to cardiovascular disease Risk factors including SBP and BMI, and negatively related to ankle-brachial index(ABI)^17^.

In a study with 6743 participants without a history of cardiovascular disease, the inter-arm SBPD of 15 mmHg and more was reported in 4.5% of participants, which was associated with the occurrence of cardiovascular disease^6^. In addition, the difference in systolic blood pressure between the arms is related to all-cause mortality and cardiovascular diseases^29^. In a prospective study of 230 hypertensive patients who were followed up for 8–9 years, inter-arm SBPD was observed in 33% of the patients, and each millimeter of increase in this difference increased the mortality rate by 5–6%^8^.

In patients without specific cardiovascular disease, every 5 mm increase in the inter-arm SBPD increases the risk of vascular events by 12%. Also, this difference is related to the occurrence of stroke in the future^20^.

Medicine in CVD patients can affect the inter-arm blood pressure difference. Miyashima et al. showed that the use of antihypertensive drugs in patients with CVD_s_ affects the inter-arm blood pressure difference but this relationship was not observed for blood lipid and blood-sugar-lowering medications^30^. In the present study, we could not consider this issue due to the missing and incompleteness of the type of medicine for CVD patients.

### Strength and limitations

In this study, the relatively large sample size ensured that the findings are to some extent representative of all Iranian adults. The cross-sectional nature of the study serves to provide evidence for the relationship between independent variables and CVD and does not establish causality. Also, due to missing information on the medicines of cardiovascular patients, we could not investigate their effect on the inter-arm blood pressure difference.

## Conclusion

Our findings suggest an association between inter-arm SBPD and CVD. After adjusting for covariates, we observed a relationship between inter-arm SBPD ≥ 15 and inter-arm DBPD ≥ 10 mm Hg with CVD. Hence, inter-arm blood pressure could be considered a marker for the prevention and diagnosis of CVD for physicians. It is worth mentioning that despite the recommendation of most guidelines to measure blood pressure in both arms, this measure has not yet been implemented in primary care. Therefore, it is necessary to measure blood pressure from both arms when measuring blood pressure for the first time, and detecting the difference in blood pressure between the arms can help in predicting the occurrence of cardiovascular diseases in the future.

## Data Availability

Data can be inquired from the corresponding author.
